# Late Cervical Cerclage at 30 Weeks of Gestation: A Case Report

**DOI:** 10.7759/cureus.94755

**Published:** 2025-10-16

**Authors:** Patricia Reyna Dueñas, Carlos Linder Efter, Andrea Olguín-Ortega

**Affiliations:** 1 Gynecology, American British Cowdray Medical Center, Mexico City, MEX; 2 Obstetrics and Gynecology, American British Cowdray Medical Center, Mexico City, MEX; 3 Gynecology, National Institute of Perinatology, Mexico City, MEX

**Keywords:** cerclage, cervical insufficiency, late cerclage, mcdonald, preterm birth

## Abstract

A 34-year-old woman was experiencing her fourth pregnancy, which was totally normal till she arrived at 29.1 weeks of gestation, beginning with uterine activity, which required hospitalization and was controlled with intravenous (IV) medication. Once discharged, in her follow-up appointment a week later, when she was 30.3 weeks, a cervical shortening was observed when the transvaginal ultrasound was made. This, in conjunction with her previous history of preterm birth, led to the decision to place a McDonald-type cerclage even when guidelines usually recommend the procedure before 28 weeks. When she was 35.5 weeks pregnant, she started with uterine activity, so it was decided to remove the cerclage and expect a natural delivery. She gave birth to a female baby without complications. What we want to highlight from the following clinical case is that it is valid to place a cerclage after week 28. Each case should be assessed individually; weeks of gestation do not appear to be an absolute contraindication, but signs of infection should be considered. Late cerclage placement should be considered in specific cases with the purpose of avoiding days at the neonatal intensive care unit, as neonatal comorbidities in well-selected cases.

## Introduction

The first cerclage was performed in 1902, on a patient with a previous second-trimester loss suggestive of cervical insufficiency [1]. The diagnosis of cervical insufficiency is challenging because of the lack of clear diagnostic criteria [2]. We understand cervical insufficiency as a situation in which the cervix is unable to retain the fetus in the absence of uterine contractions, corresponding to painless cervical dilatation, in which case it is essential to exclude any pathology that could cause cervical modifications [2-4].

The reported incidence of cervical insufficiency ranges from 0.1% to 1% but can reach 8% in women with previous pregnancy loss, who have a 50% risk of preterm labor and a 10% risk of perinatal mortality [4,5]. Some tests have been described as indicating cervical insufficiency, such as “imaging of ballon traction on the cervix, assessment of the patulous cervix with Hegar or Pratt dilators, balloon elastance test,” though they have not been validated for this type of diagnosis, and cervical length (CL) has been considered the gold standard measure to identify high-risk patients and prevent preterm birth [2].

Despite the importance of the pathology, the diagnosis of cervical insufficiency, again, is complex owing to the lack of criteria and differences among the international guidelines for cerclage placement [5]. For example, the Society of Obstetricians and Gynecologists of Canada recommends cerclage for women with a history of three or more losses during the second trimester, while the American College of Obstetricians and Gynecologists (ACOG) recommends cerclage after a single second-trimester loss [5].

A short cervix is defined as one that measures <25 mm during the second trimester [4,6]. Some societies mention a cutoff of <20 mm in patients with less than 24 weeks of gestation and no other risk factors when considering medical treatment. There is no consensus regarding the cut-off for CL between 10 mm and 25 mm [2,7], but all of the guidelines recommend that surgical treatment should be considered for a CL of ≤10 mm [5]. The International Society of Ultrasound in Obstetrics and Gynecology (ISUOG) recommends that women with a CL between 15 and 20 mm be assessed with biochemical markers “fetal fibronectin, placental alpha macroglobulin-1, and insulin-like growth factor binding protein-1” [8].

Another cut-off parameter is that any cervical length below the 10th percentile is considered short [7]. Notably, CL tends to be stable, around 43 mm, between 14 and 28 weeks, and starts to shorten as gestation progresses [8]. The type of cervical cerclage (CC) can be categorized depending on the indications into four main categories [4,6]. The first category, “history indicated,” includes CC performed between 11 and 14 weeks of pregnancy in asymptomatic patients with one to three previous second-trimester losses, depending on the guidelines and a history of CC [2,5,9,10]. The second category, “ultrasound indicated,” includes cervical shortening of <25 mm in a transvaginal ultrasound before 24 weeks of gestation, in which case the first line of treatment is progesterone if there is no previous history of preterm birth [10,11]. The third category, “emergency or rescue cerclage,” includes dilation of the cervix that exposes the membranes, which are observed vaginally or by ultrasound [10,11]. The fourth category, “preventive,” is usually performed before pregnancy (that is, prophylactically) on patients with any history of cervical surgery, such as a cervical cone biopsy, and is performed by laparoscopy or laparotomy (also referred to as transabdominal cerclage) [10,12]. Another indication for transabdominal cerclage is a previous unsuccessful cerclage [10,12].

Among the various surgical approaches, historically, when ultrasound indicates that the procedure should be performed, the Shirodkar or McDonald cerclage techniques are most commonly used worldwide. Previous research has provided no evidence that one is better than the other. The McDonald technique involves the insertion of cerclage in the lower part of the cervix, at the cervicovaginal junction, while the Shirodkar cerclage is placed higher and requires the resection of the bladder [10-12]. On the other hand, preventive cerclage, as mentioned, involves transabdominal cerclage, allowing the placement of the suture at the internal os, which provides more structural support; this technique was first described in 1965 [12].

Absolute contraindications for any kind of CC are chorioamnionitis, fetal malformations, and uterine contractions, and fetal viability must be confirmed in all cases [10,11]. The complications have been well described, as with any surgical procedure, though they are rare. The most frequent are fistula (in this case, cervicovaginal fistula), abscess, and erosion, so the risks and benefits should be considered before the procedure is performed [13].

In post-procedure management, progesterone and perioperative tocolysis should be used with caution, for most medical societies have concluded that there is no proven benefit. The most commonly recommended agent is indomethacin, though only the ACOG and the ISUOG recommend it, and only in selective cases [5]. On the other hand, the evidence for patients who already have a CC in conjunction with vaginal progesterone is limited and differs depending on the society recommendation, with the ACOG and the ISUOG being the only societies that recommend this approach, and, again, only in specific situations [5]. Most studies that have evaluated this issue have been small and yielded variable results, so more investigation is needed [6].

Most medical societies recommend removing the CC between 36 and 37 weeks of gestation in cases of a vaginal delivery. In a planned cesarean delivery, CC can be removed after the surgery. In cases of transabdominal cerclage, the recommendation is to keep the CC and consider a cesarean delivery [11].

## Case presentation

**Figure 1:**
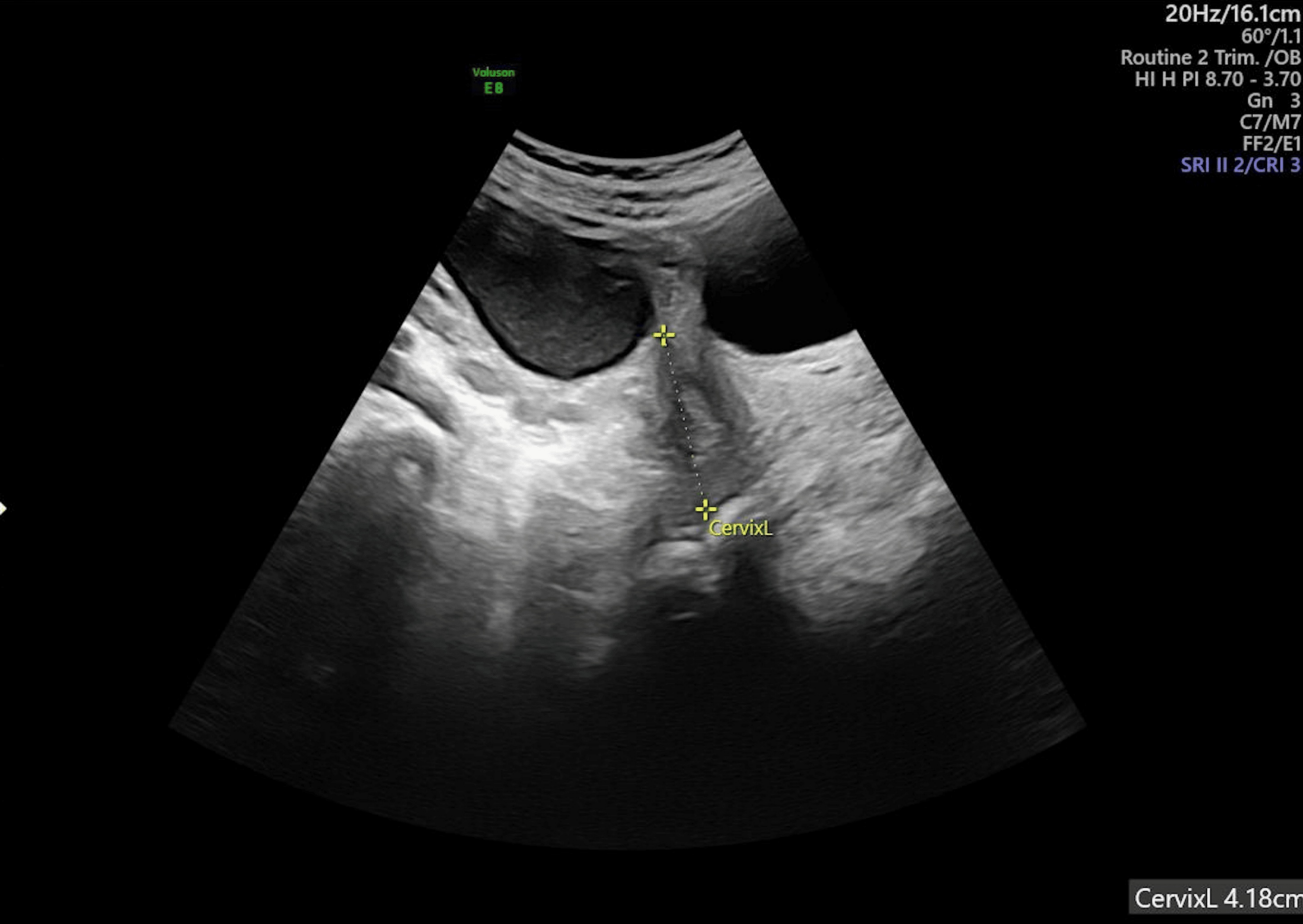
The patient’s midtrimester ultrasound at 23.1 weeks of pregnancy

The case involved a 34-year-old woman, G4, L1 (3,050 grams), L2 (3,100 grams), and L3 (1,600), with a previous premature labor and diagnosis of non-radiographic axial spondyloarthritis. She received no medication for this condition and had no other disease that causes premature delivery. Her last labor was at 33 weeks. The actual pregnancy was 30.3 weeks from her last period and evolved normally until 29.1 weeks, with normal vaginal cultures in the first trimester and no pathology in her urinary test, urine culture, or blood tests during the second trimester. She received a midtrimester ultrasound at 23.1 weeks that showed a cervical length of 41.8 mm (Figure 1).

Regular contractions began at 29.1 weeks and were associated with pain, so we performed the laboratory tests shown in Table 1 as well as vaginal culture and urinary tests, and observed no alterations. The only abnormal finding was an erythrocyte sedimentation rate of 33 mm/hr and 5% of bands; the other inflammatory reactants were normal. 

**Table 1:** Laboratory tests

Measure	Result	Parameter
Leucocytes	8.91 10ʌ3/uL	4.80–11.00 10 ʌ 3/uL
Hemoglobin	13.4 g/dl	13.5–16.5 g/dl
Hematocrit	39.2%	38.5–47.0%
Platelets	303 10ʌ 3/uL	150–450 10 ʌ 3/uL
Bands	5%	
Erythrocyte sedimentation rate	33 mm/hr	0–20 mm/hr
Protein C	0.16 mg/dl	0.00–0.50 mg/ml
Procalcitonin	0.02 ng/ml	0.00–0.50 nl7ml

Accordingly, we decided to manage uterine activity with three 100 mg indomethacin rectal suppositories combined with 30 mg nifedipine every 12 hours and to administer antenatal corticosteroid therapy for lung maturation with two doses of 12 mg intramuscular betamethasone every 24 hours in light of her previous preterm birth. Since her control of uterine activity was good, she was discharged for home care the next day. At home, the condition was managed with 200 mg vaginal progesterone every 24 hours, 100 mg piperidolate every eight hours, and 30 mg nifedipine every 12 hours, as well as office control for a week.

**Figure 2:**
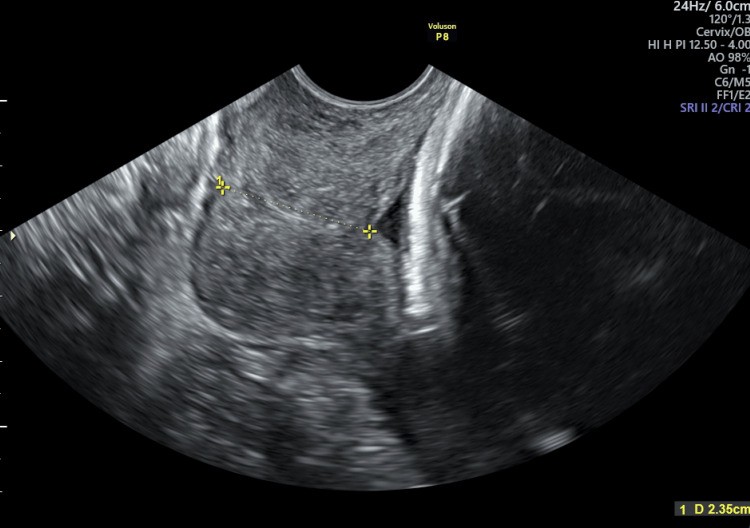
The patient’s midtrimester ultrasound at 30.3 weeks of pregnancy

A week later, at 30.3 weeks, we checked the patient’s cervical length with vaginal ultrasound and found a CL of 23.5 mm and a funneling sign (Figure 2). Therefore, we decided to perform McDonald-type CC. Her previous labs showed no sign of infection, and her inflammatory reactants were within the normal range, so we attributed the previous abnormally high results to her non-radiographic axial spondyloarthritis. 

The McDonald-type cerclage surgery was successful. A non-absorbable suture (Ethibond multifilament) was used with no complications during the procedure. The patient went home, but, at 35.5 weeks of gestation, she again experienced uterine activity, so we decided to remove the cerclage, though the recommendation for doing so is between 36 and 37 weeks, since she was near 36 weeks, and there was a risk of cervical amputation associated with the uterine activity. She delivered a 2,100-gram baby, with no need for the neonatal intensive care unit, and went home two days later.

## Discussion

A diagnosis of cervical insufficiency was made secondary to the inability to retain a pregnancy, and we ruled out other pathologies (such as vaginal and urinary infections as well as uterine activity). We considered this patient to be at high risk for preterm delivery given that her previous birth was preterm (before 34 weeks). Since preterm birth is the leading cause of neonatal mortality worldwide and has severe social and economic impacts, we decided on surgical intervention to improve the long-term outcomes for the baby, though the guidelines suggested doing so before 28 weeks. Such interventions remain controversial because there has been insufficient long-term study of neonatal outcomes within the recommended timeframe. Thus, it is important to consider as many case reports and articles as possible involving late cerclage placement before 32 weeks of gestation. We suggest 32 weeks as the cutoff since, before then, there may still be a risk of severe neonatal complications, such as interventricular hemorrhage, respiratory distress, or necrotizing enterocolitis, depending on the effectiveness of neonatal therapy, especially in low- and middle-income countries such as Mexico.

## Conclusions

The patient delivered a healthy, 2,100-gram baby at 35.5 weeks and was able to go home with the baby without a stay in the neonatal intensive care unit. Though most cerclage procedures are performed during the second trimester, before 28 weeks of gestation, we strongly suggest weighing the risks and benefits for the pregnant woman and the baby. Of course, clinical guidelines are intended to help healthcare workers make the best possible decisions, but each case must be considered individually, and the available resources must be used to achieve the best possible neonatal outcome. In our case, no fibronectin was available in the hospital, and its cost was high, so we relied on other resources for decision-making, including a physical examination, the patient’s medical history, and laboratory tests. We conclude that, even when the guidelines suggest performing cerclage before 28 weeks of gestation, the procedure can be performed later in some cases in which the failure to do so could lead to severe adverse outcomes.

## References

[1] Shennan AH, Story L (2022). Cervical cerclage: green-top guideline no. 75. BJOG.

[2] American College of Obstetricians and Gynecologists (2014). ACOG Practice Bulletin No.142: cerclage for the management of cervical insufficiency. Obstet Gynecol.

[3] Thakur M, Jenkins SM, Mahajan K (Thakur M, Jenkins SM, Mahajan K). Cervical insufficiency. https://www.ncbi.nlm.nih.gov/books/NBK525954/.

[4] Senarath S, Ades A, Nanayakkara P (2020). Cervical cerclage: a review and rethinking of current practice. Obstet Gynecol Surv.

[5] Mudrik A, Levy R, Petrecca A, Gulersen M, Chauhan SP, Erez O, Rottenstreich M (2025). Guidelines on cerclage placement: a comparative systematic review. Am J Obstet Gynecol MFM.

[6] Jones EO, Liew ZQ, Rust OA (2020). The short cervix: a critical analysis of diagnosis and treatment. Obstet Gynecol Clin North Am.

[7] Biggio J (2024). SMFM consult series #70: management of short cervix in individuals without a history of spontaneous preterm birth. Am J Obstet Gynecol.

[8] Coutinho CM, Sotiriadis A, Odibo A (2022). ISUOG Practice Guidelines: role of ultrasound in the prediction of spontaneous preterm birth. Ultrasound Obstet Gynecol.

[9] Story L, Shennan A (2024). Cervical cerclage: an evolving evidence base. BJOG.

[10] Shennan A, Story L, Jacobsson B, Grobman WA (2021). FIGO good practice recommendations on cervical cerclage for prevention of preterm birth. Int J Gynaecol Obstet.

[11] Giouleka S, Boureka E, Tsakiridis I (2023). Cervical cerclage: a comprehensive review of major guidelines. Obstet Gynecol Surv.

[12] Clark NV, Einarsson JI (2020). Laparoscopic abdominal cerclage: a highly effective option for refractory cervical insufficiency. Fertil Steril.

[13] Alani S, Wang J, Suarthana E, Tulandi T (2023). Complications associated with cervical cerclage: a systematic review. Gynecol Minim Invasive Ther.

